# Inferring potential landscapes from noisy trajectories of particles within an optical feedback trap

**DOI:** 10.1016/j.isci.2022.104731

**Published:** 2022-07-19

**Authors:** J. Shepard Bryan, Prithviraj Basak, John Bechhoefer, Steve Pressé

**Affiliations:** 1 Department of Physics, Arizona State University, Tempe, AZ, USA; 2 Department of Physics, Simon Fraser University, Burnaby, BC, USA; 3 School of Molecular Sciences, Arizona State University, Tempe, AZ, USA

**Keywords:** Physics, Optics, Statistical physics

## Abstract

While particle trajectories encode information on their governing potentials, potentials can be challenging to robustly extract from trajectories. Measurement errors may corrupt a particle’s position, and sparse sampling of the potential limits data in higher energy regions such as barriers. We develop a Bayesian method to infer potentials from trajectories corrupted by Markovian measurement noise without assuming prior functional form on the potentials. As an alternative to Gaussian process priors over potentials, we introduce structured kernel interpolation to the Natural Sciences which allows us to extend our analysis to large datasets. Structured-Kernel-Interpolation Priors for Potential Energy Reconstruction (SKIPPER) is validated on 1D and 2D experimental trajectories for particles in a feedback trap.

## Highlights

• A feedback trap was used to generate noisy Langevin microbead trajectories
• The potential energy surface is recovered using a Bayesian formulation
• The formulation uses a structured-kernel-interpolation Gaussian process (SKI-GP) to tractably approximate Gaussian process regression for larger datasets
• Thanks to our adaptation of SKI-GP, we have broadened the use of Gaussian processes for natural science applications

## Graphical abstract

**Figure:**
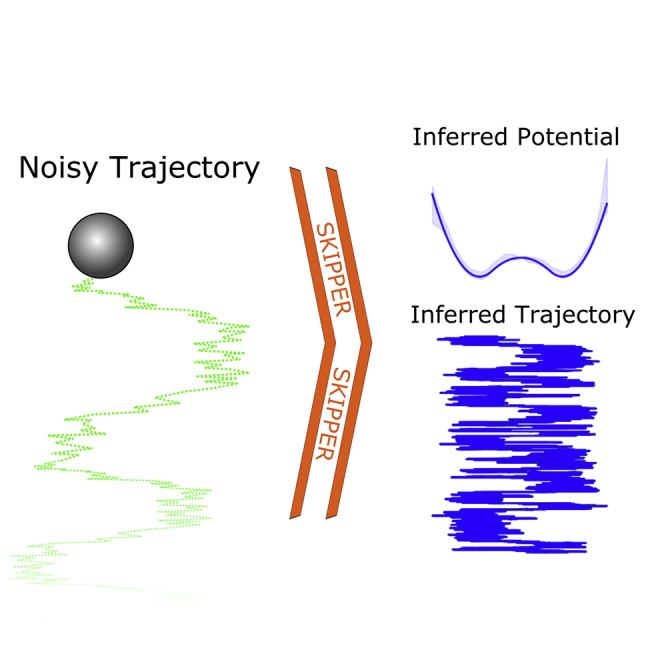

## Introduction

Determining potentials governing particle dynamics is of fundamental relevance to materials science (Deringer et al., 2019; Handle and Sciortino, 2018; Oh et al., 2022), biology (Makarov, 2015; Wang and Ferguson, 2016; Wang et al., 1997; Chu et al., 2013; Kang and Liu, 2021; Wu et al., 2021), and beyond (García et al., 2018; Dudko et al., 2006; Preisler et al., 2004; La Nave et al., 2002). For example, shapes of energy landscapes provide reduced dimensional descriptions of dynamics along reaction coordinates (Wang and Ferguson, 2016; Wang and Verkhivker, 2003; Chu et al., 2013) and key estimates of thermodynamic and kinetic quantities (Hänggi et al., 1990; Berezhkovskii et al., 2017; Bessarab et al., 2013). Shapes of energy landscapes also provide key insight into molecular function such as the periodic three-well potential of the $F_{\text{o}}F_{1}$-ATP synthase rotary motor (Wang and Oster, 1998; Toyabe et al., 2012) and the asymmetric, linearly periodic potentials responsible for kinesin’s processivity (Kolomeisky and Fisher, 2007). In a different class of applications, fundamental experimental tests of statistical physics (Proesmans et al., 2020; Wu et al., 2009) often employ potentials with deliberately complex shapes created from feedback traps based on electrical (Cohen, 2005; Gavrilov et al., 2013), optical (Kumar and Bechhoefer, 2018a; Albay et al., 2018), or thermal forces (Braun et al., 2015), or optically generated with phase masks (Hayashi et al., 2008) or spatial light modulators (Chupeau et al., 2020).

Inferring naturally occurring energy landscapes or verifying artificially created potentials demands a method free of *a priori* assumptions on the potential’s shape. This requirement rules out many commonly used methods devised for harmonic systems (Neuman and Block, 2004; Berg-Sørensen and Flyvbjerg, 2004; Jones et al., 2015; Gieseler et al., 2021) or alternative, otherwise-limited, methods to deduce potentials from data (Reif, 2009; Türkcan et al., 2012; García et al., 2018; Wang et al., 2019; Frishman and Ronceray, 2020; Yang et al., 2021; Stilgoe et al., 2021). For example, some methods (Reif, 2009; Türkcan et al., 2012) necessarily rely on binned data, relating potential energies to Boltzmann weights or average apparent force, thereby limiting the frequency of data in each bin and requiring that equilibrium be reached before data acquisition. Other methods assume stitched locally harmonic forms (García et al., 2018). Still others use neural networks (Wang et al., 2019) to deduce potentials; the uncertainty originating from measurement error and data sparsity is then not easily propagated to local uncertainty estimates over the inferred potential.

In previous work (Bryan IV et al., 2020), we introduced a method starting from noiseless one-dimensional time series data to infer effective potential landscapes without binning, or assuming a potential form, even in the case of limited sampling, while admitting full posterior inference (and thus error bars or, equivalently, credible intervals) over any candidate potentials arising from sparse data. Our method was, however, fundamentally limited to one dimension (because of the poor scaling of the computation with respect to the dataset size). As such, the method could not distinguish the effects of inherent stochasticity in the dynamics due to temperature, say, and noise introduced from the measurement apparatus. It would therefore be helpful to develop a method capable of discriminating between both noise sources and learning features of both just as the perennial hidden Markov model achieves for discrete state spaces (Rabiner and Juang, 1986; Sgouralis and Pressé, 2017).

Here, we introduce a method to infer potentials from noisy, multidimensional data often sampled in a limited fashion. We take advantage of tools from Bayesian nonparametrics to place priors on potentials assuming no prior functional forms for these potential. To do so, we introduce structured-kernel-interpolation Gaussian processes (Wilson and Nickisch, 2015) (SKI-GP) to the Natural Sciences in order to circumvent the otherwise-prohibitive computational scaling of widely used Gaussian processes. In our application, we utilize the power of SKI-GP to create Structured-Kernel-Interpolation Priors for Potential Energy Reconstruction (SKIPPER). SKIPPER can be inferred from trajectories while meeting all the following criteria simultaneously: 1) no reliance on binning or pre-processing, 2) no assumed analytic potential form, 3) inferences drawn from posteriors, allowing for spatially nonuniform uncertainties to be informed by local density of available data in specific regions of the potential (e.g., fewer data points around barriers), 4) treatment of multidimensional trajectories, 5) rigorous incorporation of measurement noise through likelihoods, and 6) compatible with lightly sampled trajectories. No other existing method meets all six criteria simultaneously.

This work serves the dual role of a method for inferring potentials with SKIPPER, as well as a demonstration of how to incorporate SKI-GPs into an inference framework. In particular, we demonstrate how to construct an effective kernel matrix for an unknown variable (the potential landscape) and how to directly sample the variable from a conditional posterior.

## Results

We benchmark SKIPPER on experimental data on a double-well potential and show that we can accurately infer the shape of the potential. We then show that SKI-GP allows us to explore 2D time series data (previously infeasible due to large amounts of data using a naive GP). We finally apply SKIPPER to trajectories in a high-barrier landscape where traces are too short to reach equilibrium. A demonstration on data from a simple harmonic well, a complicated 2D potential, and robustness tests over parameters of interest can be found in the SI (Bryan IV et al., 2022).

For testing the accuracy and effectiveness of SKIPPER, we simultaneously collected two measurements of each trajectory, one using a detector with low measurement noise and one using a second detector with higher measurement noise as shown in section. We refer to the low-noise trajectory as the “ground truth” trajectory, although it itself is subject to a small amount of measurement noise. For each experiment, we impose a potential on the particle using our feedback trap. We refer to this applied potential as the “ground truth” potential, although it may differ from the actual potential the particle experiences due to errors in the feedback trap setup, as well as experimental limitations such as drift.

### Demonstration on simulated data

**Figure 1:**
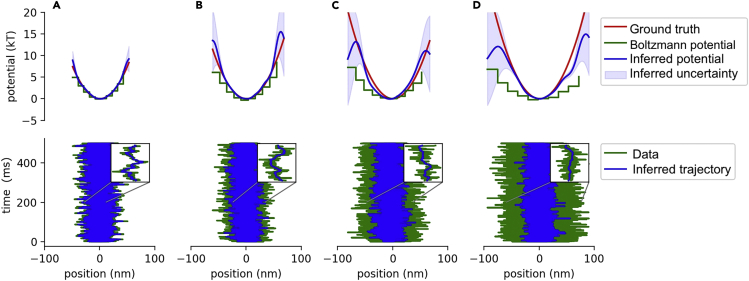
Demonstration on data from a harmonic potential Here, we analyze four datasets with increasing measurement noise. For each dataset, we plot the inferred potential in the top row along with the ground truth and the results of the Boltzmann method. We plot the inferred trajectory with uncertainty against the ground truth in the bottom row. For clarity, we zoom into a region of the trajectory (200 ms–201 ms). Measurement noise is added by increasing the optical density of the ND filter. The optical densities of the sub-figures A, B, C, and D are 0, 0.3, 0.5, and 0.6, respectively. Each trace contains 50,000 data points.

We started by analyzing simulated trajectories from a simple harmonic well. Results are shown in Figure 1. Each column shows the inferred potential (top row) and inferred trajectory (bottom row) for each dataset analyzed. We provide uncertainties and ground truth estimates for both the potential and trajectory. Additionally, for sake of comparison, we also show the potential estimated using the Boltzmann method (Bryan IV et al., 2020; Reif, 2009) (discussed in section). We highlight that the Boltzmann method does not provide trajectory estimates. By contrast, SKIPPER infers those positions obscured by noise.

Figure 1 shows that the ground truth potential and trajectory fall within our error bars (credible interval) for all datasets up to ND = 7. At ND = 7, the inferred potential develops bumps where it is unable to infer the trajectory accurately, resulting in a 2 nm shift of the potential well minimum. On the other hand, the Boltzmann method (see earlier discussion) does well in the low-noise case (Figure 1, left column) but fails as the noise introduced grows (Figure 1, right column). This is expected, as measurement noise broadens the position histogram and thereby the potential. Using SKIPPER, the estimate of the potential drops at the edges of the spatial region sampled by the particle, since the high-potential edges are rarely visited by the particle. We thus have insufficient information through the likelihood to inform those regions, and the inferred potential reverts back to the prior (set at 0, as described earlier).

**Figure 2:**
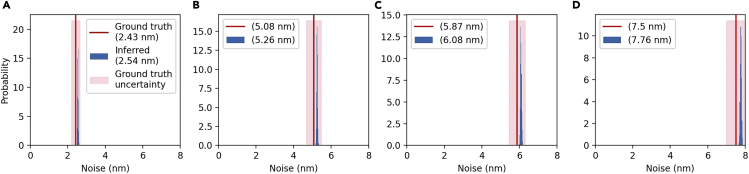
Inferred noise levels from experiments on harmonic well We analyze four datasets with increasing measurement noise. For each dataset, we plot the probability density function of the inferred magnitude of the noise against a vertical line representing the best estimate inferred using the calibration techniques outlined earlier, with a shaded pink region representing the uncertainty in the calibrations establishing “ground truth.” Measurement noise is added by increasing the optical density of the ND filter, thereby decreasing the light intensity incident on the QPD. The optical densities of sub-figures (A, B, C, and D) are 0, 0.3, 0.5, and 0.6, respectively. Each trace contains 50,000 data points.

In addition to inferring estimates for the potential energy landscape and trajectory, we also estimate the magnitude of the measurement noise for each experiment. Figure 2 shows the inferred measurement noise magnitudes obtained from SKIPPER for each single-well experiment, with the mean of our PDFs within 5% of the “ground truth” measurement, for each dataset analyzed.

Next, to show the full utility of SKIPPER, we analyzed simulated data from a complicated 2D potential with wells in the shape of a winky face. The winky face was constructed from a grid of Gaussian wells with potential energy corresponding to the pixels of a winky face. The winky face is 13 × 13 pixels, where each pixel is 10 nm wide. The eyes and mouth are about 2 kT less than the rest of the face. The white space around the face has potential energy above 20 kT, to ensure that the particle stays inside the face. We simulated a 50,000 data point trace of a particle moving along the winky face.

**Figure 3:**
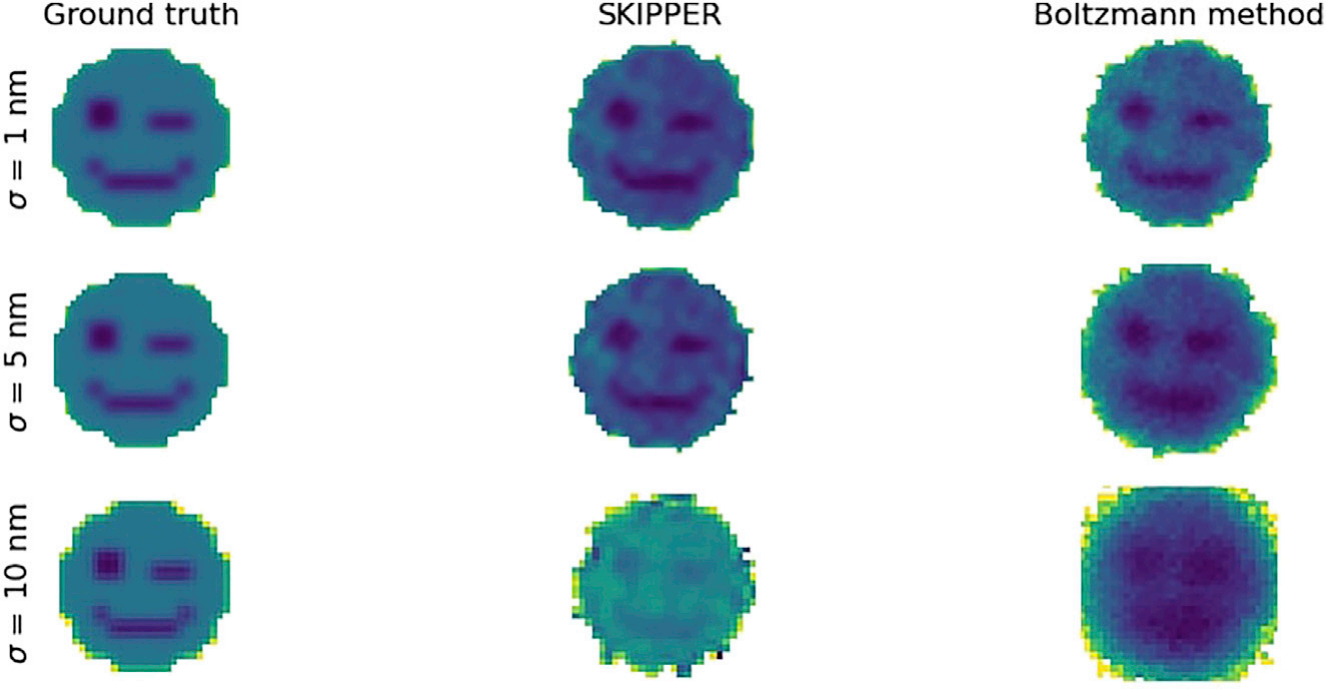
Demonstration on 2D winky face potential Here, we show results from inference on a 2D potential with wells in the shape of a winky face. The left column shows the ground truth potential energy landscape. The middle column shows inference using SKIPPER. The right column shows inference using the Boltzmann histogram method. The top row analyzes data simulated with 1 nm measurement noise. The middle row analyzes data simulated with 5 nm measurement noise. The bottom row analyzes data simulated with 10 nm measurement noise. Note that in our display pixels with potential energy greater than 5 kT are white.

Figure 3 shows results on this potential. Note that potential energies greater than 5 kT and potential energies in areas where the particle is not encountered are displayed as white background. In the top row of Figure 3, when measurement noise is small compared to the size of the face (1 vs 130 nm), both SKIPPER and the Boltzmann method are able to reconstruct a potential energy landscape that is recognizably a winky face. However, with moderate noise (σ= 5 nm), SKIPPER is able to pick up the fine detail in the face that is missed by the Boltzmann method (Figure 3 middle row). In the case of high noise (σ= 10 nm), SKIPPER’s advantage over the Boltzmann method is more dramatic, as SKIPPER’s reconstruction is still recognizably a winky face, whereas the Boltzmann method’s reconstruction not only fails to resemble a face but also overestimates the width of the face (Figure 3 bottom row).

### Robustness tests on simulated data

In order to probe SKIPPER’s robusteness with respect to parameters of interest, we demonstrate SKIPPER on data simulated with a single-well potential analyzed under different circumstances.

First, we tested SKIPPER’s robustness with respect to the number of data points on simulated data. Supplementary figure SI-1 shows the results on simulated data. Trivially, when the trajectory is so short that the particle does not travel across the well (N=50 as in Fig. SI-1 left panel), we cannot infer the shape of the potential. When the particle samples the entire well (N=500 as in Fig. SI-1 s panel), we infer the general shape of the potential, but the inference is highly impacted by small stochastic anomalies. See for example the far right of the potential, where a few higher- and lower-than-expected thermal kicks at the right side of the well caused SKIPPER to infer a second well. For reasonable inference, a few thousand points suffice (N=5000 in Fig. SI-1, third panel). When there are tens of thousands of data points (N=50,000 in Fig. SI-1), our inferred potential almost exactly overlaps with the ground truth.

We then tested SKIPPER’s robustness with respect to measurement noise in order to probe the regime in which SKIPPER can be used. Supplementary figure SI-2 shows the results. SKIPPER was robust to measurement noise for the four values of measurement noise variance chosen, but could not reproduce the potential energy landscape when the magnitude of the noise was of the same order as the maximum range of the particle. Even so, when the magnitude of the noise was up to 10% the maximum range of the particle (σ=10 nm), we nonetheless inferred the potential energy landscape accurately.

### Double well

**Figure 4:**
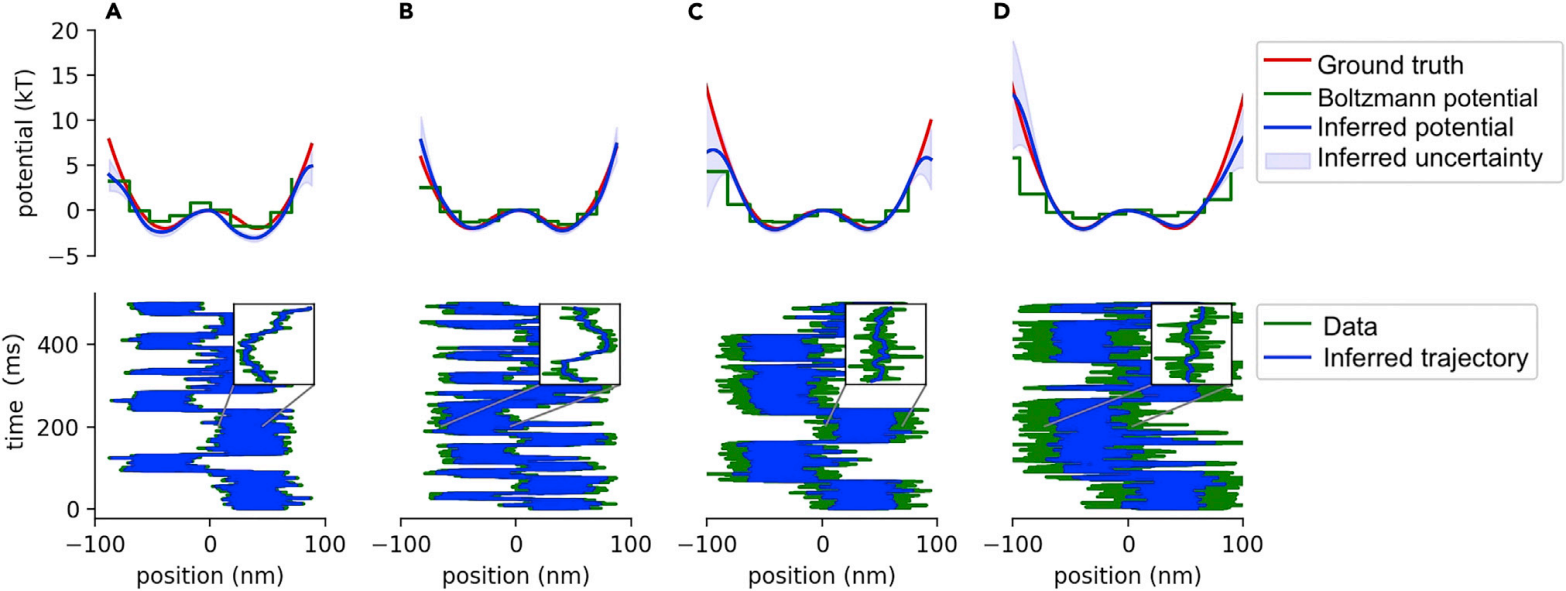
Demonstration on data from a double-well potential We analyze four datasets with increasing measurement noise. For each dataset, we plot the inferred potential in the top row alongside the ground truth and results of the Boltzmann method. We plot the inferred trajectory against the ground truth in the bottom row. For clarity, we zoom into a region of the trajectory (200 ms–201 ms). Measurement noise is added by increasing the optical density of the ND filter. The optical densities of the sub-figures (A, B, C, and D) are 0, 0.3, 0.5, and 0.7, respectively. Each trace contains 50,000 data points.

We analyzed data from a real particle in a double-well potential. Results are shown in Figure 4. Each column shows the inferred potential (top row) and inferred trajectory (bottom row) for each dataset analyzed. We provide uncertainties and ground truth estimates for both the potential and trajectory. Additionally, for sake of comparison, we also show the potential estimated using the Boltzmann method (Bryan IV et al., 2020; Reif, 2009). Briefly, the Boltzmann method, as outlined in the SI (Bryan IV et al., 2022), assumes that the bead localizations are sampled from the Boltzmann distribution and derives an estimate of the potential in discretized bins of space from the log of the relative frequency the bead is seen in each bin. We highlight that the Boltzmann method does not provide trajectory estimates. By contrast, SKIPPER infers those positions obscured by noise. Figure 4 shows that the ground truth potential and trajectory fall within the estimated range even when the measurement noise is so large that the particle is occasionally seen in the wrong well (Figure 4, top right panel). Both SKIPPER and the Boltzmann method slightly overestimate the potential of the left well at the lowest noise level, because the (short) trajectory spends too much time in the right well, leaving the left well undersampled.

### 2D single well

**Figure 5:**
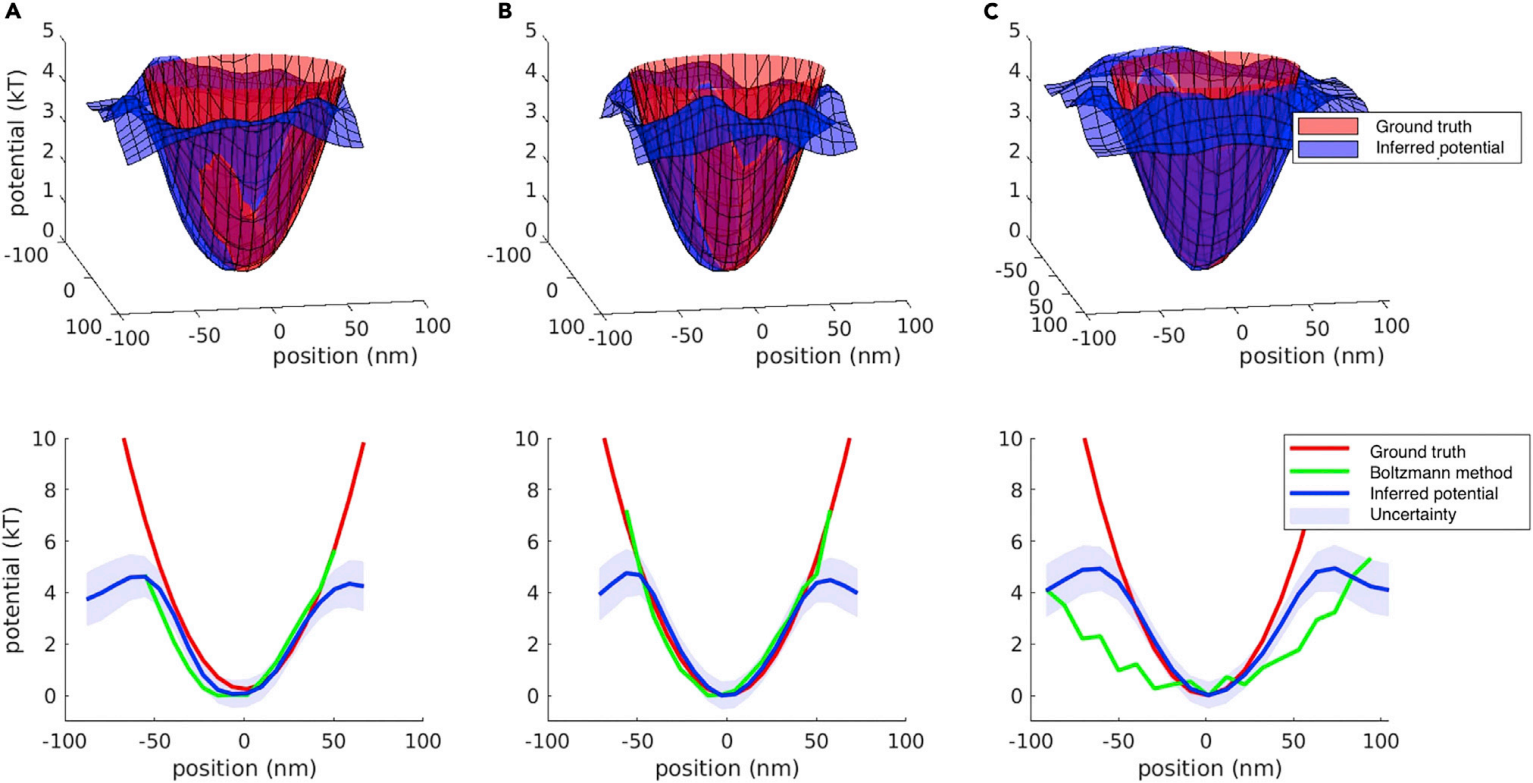
Demonstration on data from a 2D harmonic potential Top row: three datasets with increasing measurement noise. Each column shows the inferred potential results along with the ground truth potential for a different dataset. At the top, we show the inferred potential and ground truth plotted in 2D. Bottom row: 1D slice taken through the middle of the potential. Measurement noise is added by increasing the optical density of the ND filter. The optical densities of ND filter used in the sub-figures (A, B, and C) are 0.0, 0.3, and 0.7, respectively.

Next, we analyzed data from a real particle in a 2D harmonic potential. Results are shown in Figure 5. For clarity, we do not show uncertainties, trajectories, or Boltzmann-method estimates for the 2D plot, but we do show them for a 1D potential slice. Despite the added complexity in inferring the potential in full 2D at once, our estimates fall within uncertainty in regions where data are appreciably sampled, even at high measurement noise.

### Trajectories with limited sampling

One advantage of SKIPPER is that it does not rely on equilibrium assumptions. As such, we can analyze trajectories initiating from far from equillibrium conditions and with limited sampling such that the particle does not reach equillibrium in the duration of the sampling. To demonstrate this, we created real datasets where the particle starts at the top of the potential well and “rolls off” to either side. The trajectories are short (5 ms), so that the particle does not reach equilibrium during the time trace. By including the likelihoods from 100 such trajectories into our posterior, we gain information on either side of the well and can recreate the full potential, even though each individual trajectory is initiated from the top and samples only one well.

**Figure 6:**
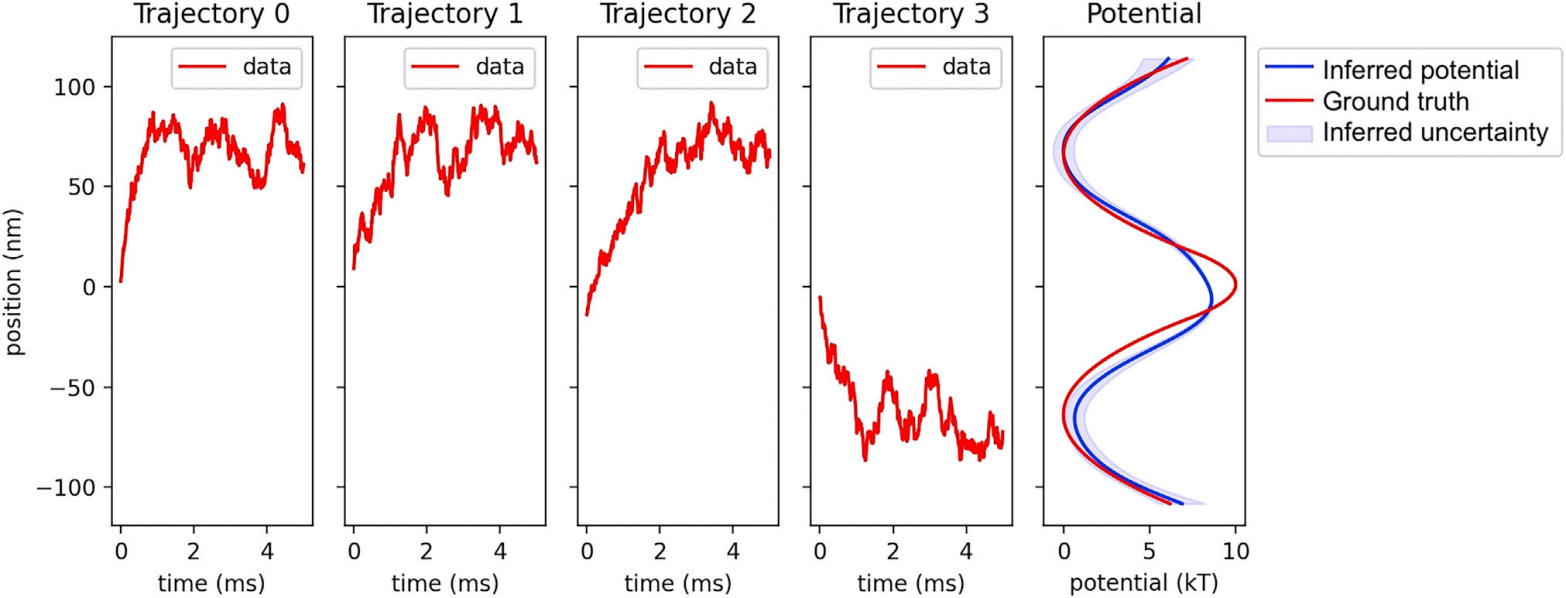
Demonstration of data from experimental trajectories with limited sampling We reconstruct a potential by analyzing many short (500 data points) trajectories with limited sampling. The left four panels show four of the 100 small data segments used to reconstruct the potential. Each trajectory starts at the top of the potential and rolls off to either side. The far right shows the inferred potential plotted with uncertainty overlaid on the ground truth potential and the potential inferred using the Boltzmann method. For comparison, the inferred and ground truth energy landscapes were shifted so that the lowest point is set to 0 kT.

In the first four panels of Figure 6, we illustrate 4 of 100 trajectories used to reconstruct the potential. We note that all trajectories start from the top of the barrier (defined as x=0), and none of the trajectories fully sample both wells.

As all trajectories start from the top of the well, we collect a disproportionally large number of data at the top of the well. Indeed, for our 10 kT barrier with roughly 50,000 samples, we would not expect to see any samples at the top of barrier in an experiment taken after the bead had reached equilibrium. Despite this extreme oversampling, SKIPPER is able to infer the height of the barrier to within 15% accuracy (SKIPPER predicts an 8.6 kT barrier; the barrier of the design potential is 10 kT). Our error bar at the top of the barrier in Figure 6 is artificially low because every trajectory initiates from the top.

## Discussion

In conclusion, inferring potential landscapes is a key step toward providing a reduced dimensional description of complex systems (Wang et al., 2019; Chmiela et al., 2017; Espanol and Zuniga, 2011; Izvekov and Voth, 2005; Manzhos et al., 2015). Here, we go beyond existing methods by providing a means of obtaining potentials, among multiple other quantities, from time series data corrupted by measurement noise. We do so by efficiently learning the potential from the rawest form of data, point-by-point. That is, we achieve this without data pre-processing (e.g., binning), assuming an analytical potential form, nor requiring equilibrium conditions. As SKIPPER is Bayesian, it allows for direct error propagation to the final estimate of the inferred potential shape. In other words, SKIPPER differs from others assuming analytic potential forms (Pérez-García et al., 2021) or projection onto basis functions (Frishman and Ronceray, 2020), as well as methods relying on neural nets (Manzhos et al., 2015) that cannot currently propagate experimental uncertainty or provide error bars reflecting the amount of data informing the potential at a particular location. Importantly, unlike the Boltzmann method (Reif, 2009), SKIPPER does not invoke any equilibrium assumption and can consider trajectories initiated from positions not sampled from an equilibrium distribution. This feature is especially relevant in studying landscapes with rarely sampled regions of space and, in particular, far from equilibrium.

SKIPPER analyzes time-independent potential energy landscapes. Realistic potentials may however vary with time (Sánchez-Sánchez et al., 2019; León-Montiel and Quinto-Su, 2017). For this reason, it may be advantageous to adapt our framework to handle time-varying potentials. This can be done by either treating the potential as a Markov process where the potential is allowed to vary slightly each frame (Williams and Rasmussen, 2006), or by extending the placement of inducing points into a time dimension, and adapting the kernel function accordingly.

In this work, we assume a Gaussian noise model, but we can, in principle, utilize different noise models by substituting Equation 4 for the desired model. As SKI-GP is general and the measurement noise model can be tuned, moving forward we could apply modified SKIPPER algorithms to map potential landscapes from force spectroscopy (Gupta et al., 2011) or even single molecule fluorescence energy transfer (Kilic et al., 2021; Sgouralis et al., 2018), with applications to inferring protein conformational dynamics or binding kinetics (Schuler and Eaton, 2008; Chung and Eaton, 2018; Sturzenegger et al., 2018; Pressé et al., 2013, 2014). In inferring smooth potentials, we would move beyond the need to require discrete states inherent to traditional analyses paradigms such as hidden Markov models (Rabiner and Juang, 1986; Sgouralis and Pressé, 2017).

Beyond inferring potentials, we believe that SKI-GPs may become a powerful tool within the Natural Sciences especially in cases, such as this one, where we deal with noisy and abundant data while learning continuous functions from data. The SKI-GP prior shifts the computational burden from inferring the potential at every data point, to inferring the potential at select inducing points allowing the computation time to scale cubically with the number of inducing points rather than cubically with the number of data points. As the number of inducing points required to create a detailed map of the potential is often far less than the number of data points, this allows for significant computational cost reduction. Furthermore, as the inducing points are static, they allow for pre-computation of the kernel matrix despite the fact that locations of the data points change at each iteration of the Gibbs sampler. As demonstrated in this work, SKI-GPs can be used to accurately and tractably map fields of variables. Such fields are ubiquitous in nature, including temperature maps (Aigouy et al., 2005), optical absorption coefficient maps (Yuan and Jiang, 2006), and diffusion coefficient maps (Taylor and Bushell, 1985; Weistuch and Pressé, 2017). We believe that this work can serve as a demonstration for how to incorporate SKI-GPs into inference frameworks that can be extended beyond potential learning.

### Limitations of the study

The model formulation requires that potentials are time independent and measurement noise is Gaussian distributed. The time scales and drag coefficient are assumed to be large enough to approximate motion with overdamped Langevin dynamics.

## STAR★Methods

### Key resources table

**Table:** 

REAGENT or RESOURCE	SOURCE	IDENTIFIER
**Software and algorithms**		

Igor Pro 9	WaveMetrics	https://www.wavemetrics.com
Numba 0.55.2	Numba	https://numba.pydata.org/
PotentialLearner	Zenodo	https://doi.org/10.5281/zenodo.6680638
Data	Zenodo	https://doi.org/10.5281/zenodo.6680673

### Resource availability

#### Lead contact

Further information and requests for resources, algorithms and methods should be directed to Steve Pressé, (spresse@asu.edu).

#### Materials availability

This study did not generate new unique materials.

### Method details

#### Experimental apparatus

The experiment is done on a modified version of the feedback optical tweezer used in various stochastic thermodynamics experiments involving virtual potentials (Kumar and Bechhoefer, 2018a, 2020). The schematics of the setup are provided in Fig. SI-3. We used a continuous-wave diode-pumped laser (HÜBNER Photonics, Cobolt Samba, 1.5 W, 532 nm) and a custom-built microscope to construct the optical trap on a vibration-isolation table (Melles Griot). We split the laser into a trapping-beam and a detection-beam using a 90:10 beam splitter. The trapping beam then passes through a pair of acousto-optic deflectors (DTSXY-250-532, AA Opto Electronic), which allows us to deflect the beam in the orthogonal XY plane. We can steer the angle of the beam and also its intensity using analog voltage-controlled oscillators (DFRA10Y-B-0-60.90, AA Opto Electronic). We increase the beam-diameter using a two-lens system in telescopic configuration to overfill the back aperture of the trapping microscope objective (MO1 in Figure S3). Then a 4f relay system images the steering point of the AOD on the back aperture of MO2. A water-immersion, high-numerical-aperture objective (MO2, Olympus 60X, UPlanSApo, NA = 1.2) is used for trapping 1.5 m diameter spherical silica beads (Bangs Laboratories) in aqueous solution (SC).

The detection beam is passed through a half-wave plate (/2), so that its polarization is orthogonal to the trapping beam, to avoid unwanted interference. It is focused using a low-numerical-aperture microscope objective (MO1, 40X, NA = 0.4) antiparallel to the trapping objective MO2. The loosely focused detection beam (compared to the trapping beam) has a larger focal spot and offers a high linear range for the position detection. We can also adjust the detection plane using a 4f relay lens system. The forward scattered detection beam from the trapped bead is collected by the trapping objective MO2 and transmitted through the polarizing beam splitter (PBS). The PBS reflects the trapping beam and thus separates the detection beam. We also place another linear polarizer (P) after the PBS to minimize the amount of leaked trapping laser. The detection beam is then separated using a beam splitter on a pair of quadrant photodiodes (QPD, First Sensor, QP50-6-18u-SD2). We control the intensity of the detection beam on QPD1 by placing a neutral density filter (ND) of desired strength and thus control the signal-to-noise ratio (SNR) on QPD1.

A red LED (660nm, Thorlabs, M660L4) illuminates the trapped particle for imaging onto a camera. The light from the LED enters the detection object through a long-pass filter (LPF, cutoff wavelength 585 nm, Edmond Optics) which reflects the detection beam. The collected illumination light is separated by a short-pass filter (SPF, cutoff wavelength 600 nm, Edmond Optics) and reflected onto a camera (FLIR BFS-U3-04S2M−CS).

Using a LabView program, we digitize the analog signal from the pair of QPDs by a field programmable gate array (FPGA, National Instruments, NI PCIe-7857). The voltage signals are then calibrated as position signals using a QPD-AOD-camera method (Kumar and Bechhoefer, 2018b). The FPGA runs the control loop and uses a programmed feedback rule to send the appropriate control signal to the AODs.

#### Measurement noise

Here we discuss the estimation of measurement noise for our experimental setup. Briefly, we obtain an estimate of the noise by recording two simultaneous trajectories of a particle and finding the difference between the trajectories. As both measured trajectories should be equal in the limit of zero noise, we can interpret any deviation between the measured trajectories as coming from noise.

We start by applying a triangle wave voltage of frequency 1.6 Hz to the AOD, to move a bead linearly along the x axis. We record the beads motion at 100 kHz using two simultaneous detectors giving output $x_{1}\left(t\right)$ and $x_{2}\left(t\right)$, respectively. We demonstrate this idea in Figure S4A. Figure S4B shows that the difference $x_{1}-x_{2}$ follows a Gaussian distribution. The mean of the Gaussian distribution shown in Fig. SI-4B, has a nonzero value of 0.28±0.02 nm, which arises from nonlinear calibration errors in the two detectors. In Figure S4C, we verify that the autocorrelation is flat, indicating that each position measurement has independent measurement noise.

To calculate the variance of the measurement noise, we denote the SD of the detectors $\sigma_{1}$ and $\sigma_{2}$, respectively. For Gaussian distributed measurement noise $x_{1}-x_{2}$ is also Gaussian, with variance equal to $\sigma_{x_{1}}^{2}+\sigma_{x_{2}}^{2}$.

We can fit the power spectral density of $x_{1}-x_{2}$ with the aliased Lorentzian expression for discretely sampled times series (Berg-Sørensen and Flyvbjerg, 2004). Because the measurement noise is Gaussian, we add a noise term to the fitting function. Thus we can estimate the noise of each trajectory by integrating the noise term over the frequency domain. From this, we estimate standard deviations of noise in $x_{1}$ and $x_{2}$ as 3.1±0.3 nm and 3.8±0.4 nm, respectively.

#### Data acquisition

We performed experiments using a feedback optical tweezer, whose details are given in section and have been described in previous work (Kumar and Bechhoefer, 2018a). Briefly, we trap a silica bead of 1.5 m diameter using an optical tweezer, which creates a harmonic well without feedback. By applying feedback, we change the shape of the potential to a double well along one of the axes. We measure the position of a bead with two different quadrant photodiodes (QPD) simultaneously to give us two trajectories ($x_{1}$ and $x_{2}$) with two different values of signal-to-noise (SNR) as explained in the section). One detector has high SNR and is used for feedback to create the desired virtual potential (Jun and Bechhoefer, 2012); the other has an adjustable SNR and is used to explore inferences from measured signals with lower SNR. We reduce the SNR in the other detector by placing neutral density (ND) filters of increasing optical density (OD) in front of it. Thus, we can use SKIPPER on the same trajectory over two different experimental SNRs and compare performance. We estimate the measurement noise and SNR in each detector from the noise floor of the power spectrum (see section).

### Quantification and statistical analysis

Concretely, our goal is to use noisy positional measurements, $y_{1:N}$, to infer all unknowns: 1) the potential at each point in space, U(⋅) (with U(x) denoting the potential evaluated at *x*); 2) the friction coefficient, ζ; 3) the magnitude of the measurement noise, $\sigma^{2}$ (under a Gaussian noise model); and 4) the actual position at each time, $x_{1:N}$. Toward achieving our goal, we construct a joint posterior probability distribution over all unknowns. As our posterior does not admit an analytic form, we devise an efficient Monte Carlo strategy to sample from it.

#### Dynamics

We describe the dynamics of the particle with an overdamped Langevin equation (Zwanzig, 2001),

(Equation 1a) $$\zeta \dot{x}\;=\;f\left(x\right)+r\left(t\right)$$

(Equation 1b) f(x)=−∇U(x),

where x(t) is the possibly multidimensional position coordinate at time *t*; $\dot{x}\left(t\right)$ is the velocity; f(x) is the force at position x(t); and ζ is the friction coefficient. The forces acting on the particle include positional forces f(x) expressed as the gradient of a conservative potential, f(x)=−∇U(x). The stochastic (thermal) force, r(t), is defined as follows:

(Equation 2a) (r(t))=0

(Equation 2b) $$\left\langle r_{i}\left(t\right)r_{j}\left(t^{\prime }\right)\right\rangle =2\zeta kT\delta \left(t-t^{\prime }\right)\delta_{ij}$$

where ⟨⋅⟩ denotes an ensemble average over realizations, *T* is the temperature of the bath and *k* is Boltzmann’s constant. Under a forward Euler scheme (LeVeque, 2007) for Equation (1a) with time points given by $t_{n}=n\Delta t$, each position, given its past realization, is sampled from a normal distribution

(Equation 3) $$x_{n+1}|x_{n},f\left(\cdot \right),\zeta \;∼\text{N}\left(x_{n}+\frac{\Delta t}{\zeta }f\left(x_{n}\right),\gamma^{2}I\right).$$

In words, “the position $x_{n+1}$ given quantities $x_{n},f\left(\cdot \right),$ and ζ is sampled from a Normal distribution with mean $x_{n}+\frac{\text{Δ}t}{\zeta }f\left(x_{n}\right)$ and variance $\gamma^{2}=\frac{2\text{Δ}tkT}{\zeta }I$.”

As is typical for experimental setups, we use a Gaussian noise model and write

(Equation 4) $$y_{n}|x_{n},\sigma^{2}∼\text{N}\left(x_{n},\sigma^{2}I\right).$$

In words, the above reads “$y_{n}$ given quantities $x_{n},\sigma^{2}$ is drawn from a normal.” Here $\sigma^{2}$ is the measurement noise variance. In Equation (4), the measurement process is instantaneous, i.e., assumed to be faster than the dynamical time scales. Our choice of Gaussian measurement model here can be modified at minimal computational cost (e.g., (Hirsch et al., 2013)) if warranted by the data, provided the final measurement noise model is stationary and each measurement depends only on the position at that time level.

#### Probabilities

Next, from the product of the likelihood ($P\left(y_{1:N}|U\left(\cdot \right),\zeta ,x_{1:N},\sigma^{2}\right)$) and the prior ($P\left(U\left(\cdot \right),\zeta ,x_{1:N},\sigma^{2}\right)$), we obtain the posterior over all unknowns

(Equation 5) $$P\left(U\left(\cdot \right),\zeta ,x_{1:N},\sigma^{2}|y_{1:N}\right)\;\propto \;P\left(y_{1:N}|U\left(\cdot \right),\zeta ,x_{1:N},\sigma^{2}\right)P\left(U\left(\cdot \right),\zeta ,x_{1:N},\sigma^{2}\right).$$

The likelihood is derived from the noise model provided in Equation (4). By contrast, the prior is informed by the Langevin dynamics, as we see by decomposing it as follows:

(Equation 6) $$P\left(U\left(\cdot \right),\zeta ,x_{1:N},\sigma^{2}\right)\;=\;P\left(x_{2:N}|x_{1},U\left(\cdot \right),\zeta \right)P\left(x_{1}|U\left(\cdot \right),\zeta \right)\left(U\left(\cdot \right)\right)P\left(\zeta \right)P\left(\sigma^{2}\right).$$

The first term on the right-hand side of Equation (6) follows from Equation (Equation 1a), (Equation 1b), while we are free to choose the remaining priors, P(U(⋅)), $P\left(x_{1}|U\left(\cdot \right),\zeta \right)$, P(ζ), and $P\left(\sigma^{2}\right)$.

Important considerations dictate the prior on the potential. First, the potential may assume any shape (and, as such, is modeled nonparametrically) although it should be smooth (i.e., spatially correlated). A Gaussian process (GP) prior (Williams and Rasmussen, 2006) allows us to sample continuous curves with covariance provided by a pre-specified kernel. However, naive GP prior implementations are computationally prohibitive, with time and memory requirements scaling as the number of data points cubed (Bryan IV et al., 2020; Williams and Rasmussen, 2006).

These size-scaling issues can be resolved by adopting an SKI-GP (Wilson and Nickisch, 2015; Wilson et al., 2015; Titsias, 2009; Gal and van der Wilk, 2014) prior for the potential, U(⋅). The SKI-GP prior is a hierarchical structure, where the potential at all points is interpolated according to *M* chosen inducing points at fixed locations, $x_{1:M}^{u}$, and where the values of the potential at the inducing points are themselves drawn from a GP, as seen in section.

We note that under this model, we shift the focus from inferring U(⋅) to inferring $u_{1:M}$ from which we recover U(⋅) and f(⋅) with a modified kernel matrix (see section).

Choices for priors on $x_{1}$, ζ and $\sigma^{2}$ are less critical and chosen for computational convenience alone. For $P\left(x_{1}|U\left(\cdot \right),\zeta \right)$, we are free to choose any prior and select

(Equation 7) $$P\left(x_{1}|U\left(\cdot \right),\zeta \right)=N\left(x_{1};0,{\text{Θ}}^{2}\right).$$

For the friction, ζ, we select a gamma distribution for which support on the positive real axis is assured. That is,

(Equation 8) $$\zeta \;∼\text{Gamma}\left(\alpha_{\zeta },\beta_{\zeta }\right).$$

Lastly, for the noise variance, $\sigma^{2}$, we place an inverse-gamma prior

(Equation 9) $$\sigma^{2}∼\text{InvGamma}\left(\alpha_{\sigma^{2}},\beta_{\sigma^{2}}\right).$$

The inverse-gamma prior is chosen because it is conjugate to the likelihood, meaning that we may directly sample from the posterior constructed by the prior multiplied by the likelihood (Bishop, 2006). The variables, Θ, $\alpha_{\zeta }$, $\beta_{\zeta }$, $\alpha_{\sigma^{2}}$, and $\beta_{\sigma^{2}}$ are hyperparameters that we are free to choose, and whose impact on the ultimate shape of the posterior reduces as more data are collected (Gelman et al., 2013; Sivia and Skilling, 2006).

#### Modified kernel matrix

Here we show how to calculate the force at an arbitrary test location using the structured kernel interpolation Gaussian process. We first derive the force for one-dimensional potentials exactly and then briefly explain how to generalize to higher dimensions. In order to calculate the force given $u_{1:M}$, we write

(Equation 10a) $$f\left(x\right)\;=-\frac{d}{dx}U\left(x\right)$$

(Equation 10b) $$\;=-\frac{d}{dx}K^{†}K^{-1}u_{1:M}$$

(Equation 10c) $$\;=K^{*}K^{-1}u_{1:M}$$

(Equation 10d) $$K^{*}\;=-\frac{d}{dx}K^{†}$$

(Equation 10e) $$K_{ij}^{\text{∗}}\;=-\frac{d}{dx}K\left(x,x_{m}^{u}\right)$$

(Equation 10f) $$\;=\frac{h^{2}}{\ell^{2}}\left(x-x_{m}^{u}\right)\text{exp}\left(-\frac{1}{2}{\left(\frac{x-x_{m}^{u}}{\ell }\right)}^{2}\right)\;,$$

where $K^{*}$ is now the covariance between the potential evaluated at the inducing points and the force evaluated at a test location, and we have assumed the kernel follows the familiar squared exponential form (Williams and Rasmussen, 2006).

To generalize to higher dimensions, we will need to calculate the force in each dimension separately. For example, to find the force in the *k* direction at a test point, x,

(Equation 11) $$f^{k}\left(x\right)=-\frac{d}{dx^{k}}U\left(x\right)$$

(Equation 12) $$\;=-\frac{d}{dx^{k}}K^{†}K^{-1}u_{1:M}$$

(Equation 13) $$\;=K^{*k}K^{-1}u_{1:M}$$

(Equation 14) $$K_{ij}^{\text{∗}k}\;=\frac{h^{2}}{\ell^{2}}\left(x^{k}-x_{m}^{u,k}\right)\text{exp}\left(-\frac{1}{2}{\left|\frac{x-x_{m}^{u}}{\ell }\right|}^{2}\right).$$

#### Inference

As our posterior does not assume an analytical form, we devise an overall Gibbs sampling scheme (Bishop, 2006) to draw samples from it. Within this scheme, we start with an initial set of values for the parameters ($\zeta^{\left(0\right)},\sigma^{\left(0\right)},x_{1:N}^{\left(0\right)},U{\left(\cdot \right)}^{\left(0\right)}$) and then iteratively sample each variable holding all others fixed (Geman and Geman, 1984). Here, we show the conditional probabilities used for our Gibbs sampling algorithm.

#### Positions

The distribution for $x_{1:N}$ is simple. If we sample each $x_{n}$ one at a time,

(Equation 15) $$\begin{array}{c} P\left(x_{1}|u_{1:M},x_{2:N}\right)\;\propto \text{N}\left(x_{1};0,\frac{2\tau kT}{\zeta }\right)\\ \;\times \;N\left(x_{2};x_{1}+\frac{\tau }{\zeta }f\left(x_{1}\right),\frac{2\tau kT}{\zeta }\right)\\ \;\times \;N\left(y_{1};x_{1},\sigma^{2}\right) \end{array}$$

(Equation 16) $$\begin{array}{c} P\left(x_{n}|u_{1:M},x_{1:n-1}\right)\;\propto \text{N}\left(x_{n};x_{n-1}+\frac{\tau }{\zeta }f\left(x_{n-1}\right),\frac{2\tau kT}{\zeta }\right)\\ \;\times \;N\left(x_{n+1};x_{n}+\frac{\tau }{\zeta }f\left(x_{n}\right),\frac{2\tau kT}{\zeta }\right)\\ \;\times \;N\left(y_{n};x_{n},\sigma^{2}\right) \end{array}$$

(Equation 17) $$\begin{array}{c} P\left(x_{N}||u_{1:M},x_{1:N-1}\right)\;\propto \text{N}\left(x_{N};x_{N-1}+\frac{\tau }{\zeta }f\left(x_{N-1}\right),\frac{2\tau kT}{\zeta }\right)\\ \;\times \;N\left(y_{N};x_{N},\sigma^{2}\right). \end{array}$$

Although these equations look Gaussian, they are not (except the last one), since we have to remember that $f_{n}=f\left(x_{n}\right)$ is a function of $x_{n}$. Thus, direct sampling is not possible, and we can only sample positions using a Metropolis-Hastings algorithm (Bishop, 2006).

#### Potential

Following the logic outlined in our previous manuscript (Bryan IV et al., 2020), we can infer the potential. For now, we focus on one-dimensional data.

We fully write the prior on positions, $x_{2:N}|U\left(\cdot \right),x_{1},\zeta$ as

(Equation 18) $$P\left(x_{2:N}|f\left(\cdot \right),x_{1}\right)\;=\text{N}\left(\tau v_{1:N-1};\frac{\tau }{\zeta }f_{1:N-1},\frac{2\tau kT}{\zeta }I\right)$$

(Equation 19) $$\;\propto \text{N}\left(f_{1:N-1};\zeta v_{1:N-1},\frac{2\zeta kT}{\tau }I\right)$$

where $f_{n}=f\left(x_{n}\right)$ and $v_{n}=\left(x_{n+1}-x_{n}\right)/\tau$. Substituting in Equation 10d, we get,

(Equation 20) $$\;=\text{N}\left(K^{*}K^{-1}u_{1:M};\zeta v_{1:N-1},\frac{2\zeta kT}{\tau }I\right)$$

(Equation 21) $$\;\propto \text{exp}\left(-\frac{1}{2}{\left(\zeta v_{1:N-1}-K^{*}K^{-1}u_{1:M}\right)}^{T}\frac{\tau }{2\zeta kT}I\left(\zeta v_{1:N-1}-K^{*}K^{-1}u_{1:M}\right)\right)$$

(Equation 22) $$\;\propto \text{exp}\left(-\frac{1}{2}\frac{\tau }{2\zeta kT}u_{1:M}^{T}K^{-1}K^{{*}^{T}}K^{*}K^{-1}u_{1:M}+\frac{1}{2}\frac{2\tau }{2kT}v_{1:N-1}^{T}K^{*}K^{-1}u_{1:M}\right)$$

(Equation 23) $$\;\propto \text{exp}\left(-\frac{1}{2}{\left(u_{1:M}-M^{-1}b\right)}^{T}M\left(u_{1:M}-M^{-1}b\right)\right)$$

(Equation 24) $$\;\propto \text{N}\left(u_{1:M};M^{-1}b,M^{-1}\right)$$

where we have used matrix completing the square, with

(Equation 25) $$b\;=\frac{\tau }{2kT}K^{-1}K^{{*}^{T}}v_{1:N-1}$$

(Equation 26) $$M\;=\frac{\tau }{2\zeta kT}K^{-1}K^{{*}^{T}}K^{*}K^{-1}.$$

Combining with the prior, we get

(Equation 27) $$P\left(u_{1:M}|x_{1:N}\right)\;\propto \text{N}\left(u_{1:M};M^{-1}b,M^{-1}\right)N\left(u_{1:M};0,K\right)$$

(Equation 28) $$\;\propto \text{N}\left(u_{1:M};{\left(K^{-1}+M\right)}^{-1}\left(K^{-1}0+MM^{-1}b\right),{\left(K^{-1}+M\right)}^{-1}\right)$$

(Equation 29) $$\;=\text{N}\left(u_{1:M};\tilde{\mu },\tilde{K}\right)$$

(Equation 30) $$\tilde{\mu }\;=\frac{\tau }{2kT}{\left(K^{-1}+\frac{\tau }{2\zeta kT}K^{-1}K^{{*}^{T}}K^{\text{∗}}K^{-1}\right)}^{-1}K^{-1}K^{{*}^{T}}v_{1:N-1}$$

(Equation 31) $$\tilde{K}\;={\left(K^{-1}+\frac{\tau }{2\zeta kT}K^{-1}K^{{*}^{T}}K^{*}K^{-1}\right)}^{-1}.$$

For multidimensional trajectories, we separate the likelihood into the forces experienced along each trajectory,

(Equation 32) $$P\left(x_{2:N}|f\left(\cdot \right),x_{1}\right)\;\propto \overset{D}{\underset{d}{\prod }}N\left(f_{1:N-1}^{d};\zeta v_{1:N-1}^{d},\frac{2\zeta kT}{\tau }I\right),$$

where *d* is the dimension index and *D* is the number of dimensions. A convenient choice of indexing allows us to sample the potential directly, even with multidimensional trajectories. It consists of “flattening out” our multidimensional force, velocity and kernel arrays into one-dimensional vectors, and “flattening out” the multidimensional kernel matrices into a matrix.

The convenient choice is as follows: Let the first *N* indices of all variables be reserved for values concerning the *N* measurements in the first dimension, then the N+1 through 2N indices be reserved for values concerning the *N* measurements in the second dimension, and continue this pattern until all D×N measurements have been accounted for. For example, define f to be a vector such that $f_{1}$ is the force at the first time level along the first dimension, $f_{2}$ is the force at the first time level along the second dimension, …, $f_{D+1}$ is the force at the first time level along the second dimension, $f_{D+2}$ is the force at the second time level along the second dimension, and so forth. By utilizing this pattern for f, v, and $K^{\text{∗}}$, we can sample the potential directly using Equation (29).

#### Friction coefficient

The conditional probability for the friction coefficient is the product of Equations (3), (7) and (8)

(Equation 33) $$\begin{array}{c} P\left(\zeta |x_{1:N},U\left(\cdot \right)\right)\;\propto \text{Gamma}\left(\zeta ;\alpha_{\zeta },\beta_{\zeta }\right)N\left(x_{1};0,\frac{2\tau kT}{\zeta }\right)\\ \;\times \;\overset{N}{\underset{n=2}{\prod }}N\left(x_{n};x_{n-1}+\frac{\tau }{\zeta }f\left(x_{n-1}\right),\frac{2\tau kT}{\zeta }\right)\;, \end{array}$$

which cannot be simplified into any known elementary distribution. We therefore sample, ζ using a Metropolis Hastings algorithm (Bishop, 2006).

#### Measurement noise

As our inverse gamma prior for $\sigma^{2}$, Equation (9) is conjugate to our likelihood, our conditional probability for $\sigma^{2}$ can be simplified to an inverse gamma distribution (Gelman et al., 2013; Sivia and Skilling, 2006)

(Equation 34) $$P\left(\sigma^{2}|y_{1:N},x_{1:N}\right)\;=\text{InvGamma}\left(\alpha_{\sigma^{2}}+\frac{N}{2},\beta_{\sigma^{2}}+\frac{1}{2}\sum {\left|x_{n}-y_{n}\right|}^{2}\right).$$

#### Boltzmann method

In the Results section, we compare to the Boltzmann method (Reif, 2009). The Boltzmann method, as opposed to the other existing methods, has the advantage that it is physically intuitive (it is derived from thermodynamics). It does not assume a potential shape *a priori* and therefore can be used for non-harmonic potentials. Its main limitation, in not treating measurement noise, is a limitation of all other competing methods.

The Boltzmann method uses the Boltzmann distribution from thermodynamics, which relates the potential in a region of space to the fraction of time that the particle will be seen in that region,

(Equation 35) $$U_{i}\;\propto \;-kT\;\log\left(p_{i}\right)$$

where $U_{i}$ is the potential of region *i* and $p_{i}$ is the fraction of the time that the particle spent in region *i*.

A limitation of the Boltzmann method is that the presence of measurement noise implies that the fraction of time that a particle *was seen* in a region, $p_{i}$, does not equal the fraction of time that the particle *was in* the region. As a consequence, measurement noise will smear the shape of the inferred potential over the range of the measurement noise (see Figure 5).

## Data Availability

Data and code are available via Zenodo. Data can be found at https://doi.org/10.5281/zenodo.6680673. Code can be found at https://doi.org/10.5281/zenodo.6680638. Any additional information required to reanalyze the data reported in this work paper is available from the Lead contact upon request.
