# Supplementary material for: Inferring potential landscapes from noisy trajectories of particles within an optical feedback trap
Source: iScience. 2022 Jul 19;25(9):104731. doi: 10.1016/j.isci.2022.104731 (PMC9400092; doi:10.1016/j.isci.2022.104731)
Supplement: Document S1. Figures S1–S4 [file mmc1.pdf]

iScience, Volume *25*

## Supplemental information

# Inferring potential landscapes from noisy trajectories of particles within an optical feedback trap

**J. Shepard Bryan IV, Prithviraj Basak, John Bechhoefer, and Steve Pressé**

# Supplementary Figures

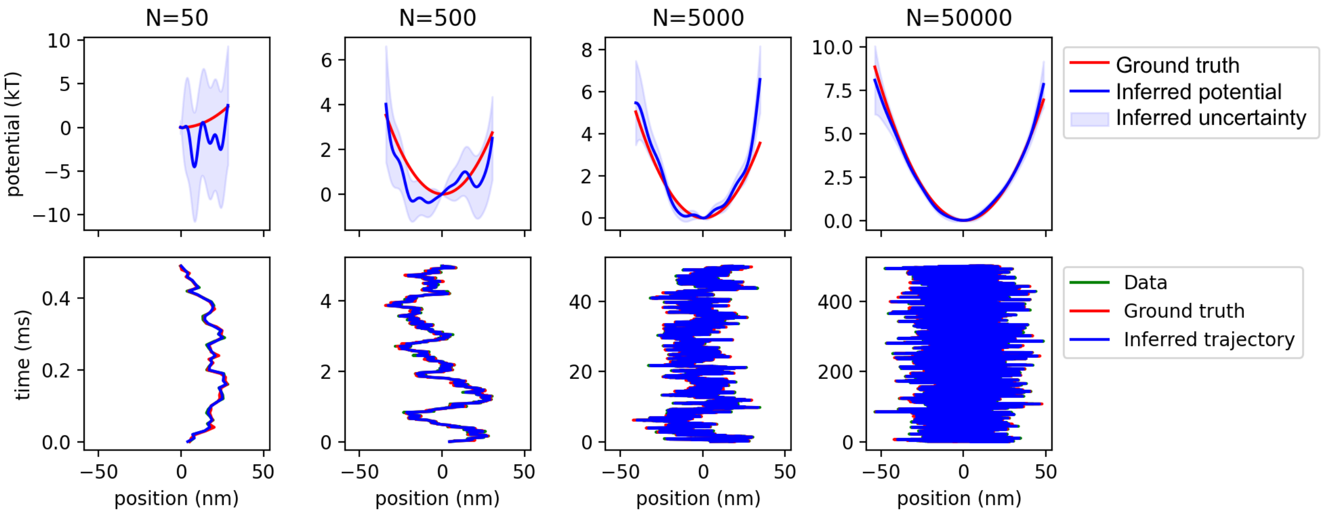


FIG. SI-1. Robustness test with respect to number of data points on real data, related to Robustness tests in Results. Here we replicate our robustness test with respect to number of data points on real data by analyzing an experimental data set with cropped at different time levels. Note that the y axis of each panel in the second row is different and corresponds to the length of the inferred trajectory.

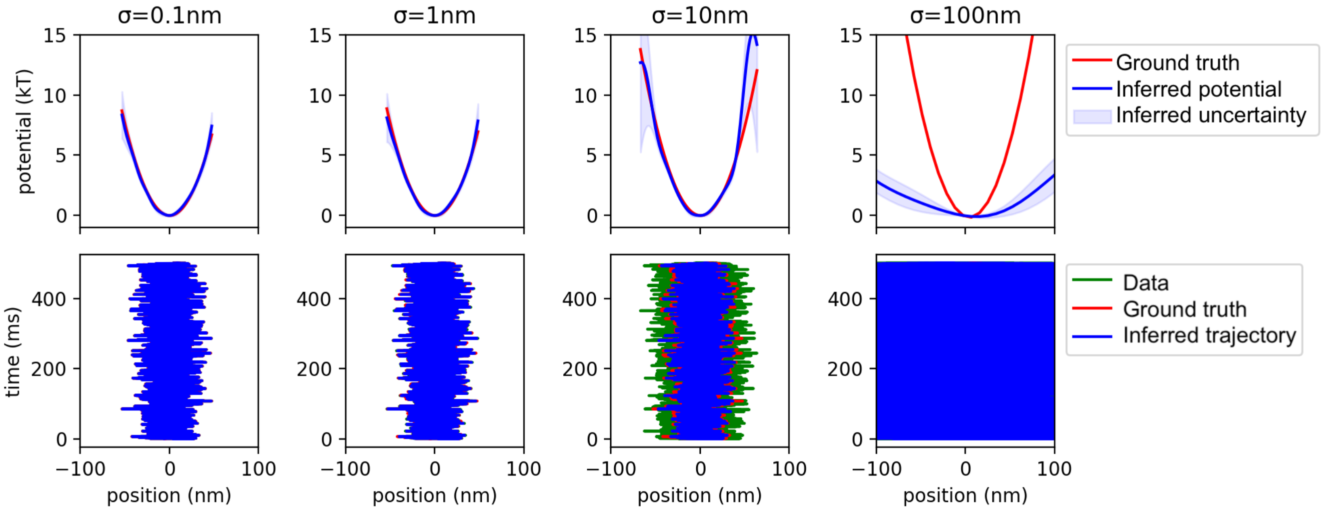


FIG. SI-2. Robustness test with respect to noise level, related to Robustness tests in Results. Here we analyze four data sets with different measurement noise magnitudes. We show that SKIPPER is robust with respect to measurement noise.

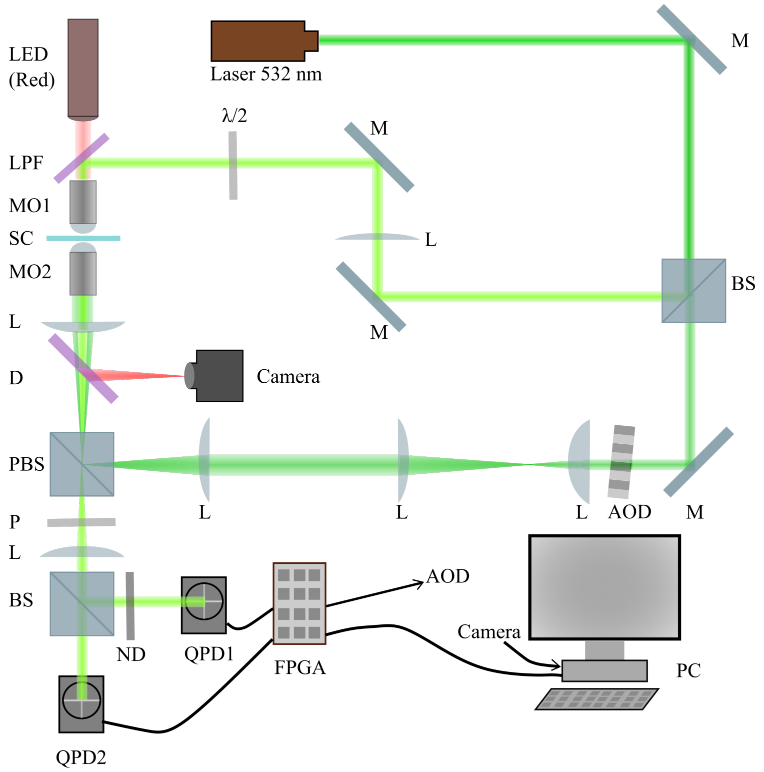


FIG. SI-3. Schematic diagram of the experimental apparatus, related to Star Methods. M = mirror, L = lens, BS = beam splitter (non-polarizing), PBS = polarizing beam splitter, AOD = acousto-optic deflector, LPF = long-pass filter, MO = microscope objective, SC = sample chamber, SPF = short-pass filter, ND = neutral density filter, QPD = quadrant photodiode, λ/2 = half-wave plate, P = linear polarizer.

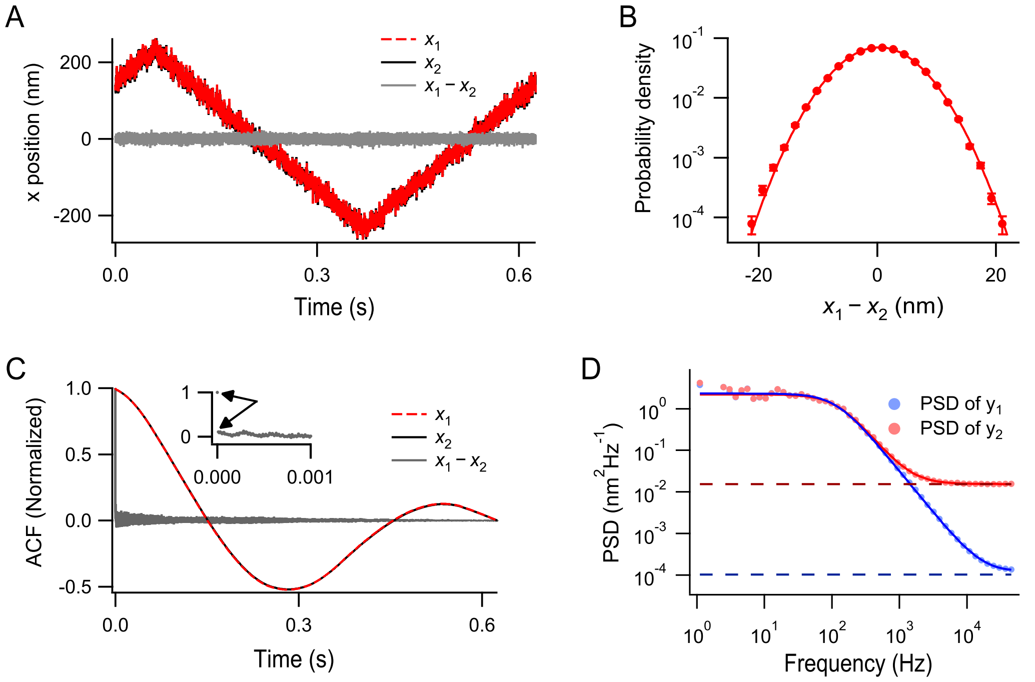


FIG. SI-4. Measurement-noise calibration, related to Star Methods. A. The response of a bead's triangle wave motion is recorded as $\boldsymbol{x}_1$ and $\boldsymbol{x}_2$ by the two detectors. If the two detectors are accurately calibrated, the difference between the two signals $\boldsymbol{x}_1 - \boldsymbol{x}_2$ in effect sums their noise. B. Histogram of the difference in the signal between the two detectors. The solid line is a fit of the data to a normal distribution, with mean $0.28 \pm 0.02$ nm and standard deviation $5.69 \pm 0.02$ nm. C. Normalized autocorrelation function of the two signal and their difference. As seen in the inset, the autocorrelation of $\boldsymbol{x}_1 - \boldsymbol{x}_2$ decays to the residual correlation in one time step. D. Estimation of noise in each of $\boldsymbol{y}_1$, $\boldsymbol{y}_2$ trajectory by fitting the corresponding power spectral density. $\boldsymbol{y}_1$ and $\boldsymbol{y}_2$ are the signals of a bead trapped in harmonic potential recorded using the two detectors at different signal to noise ratio (SNR). The solid lines are the fits to the PSD using the aliased Lorentzian formula with an added noise term. The dashed lines are the estimated noise in each of the PSD. We find the variance of the noise by integrating the noise term over the entire frequency region and estimate their signal-to-noise ratios to be 95.5 and 1.7, respectively.
